# Congenital Torticollis in a Child With Cervical Spine Deformity: A Case Report and Literature Review

**DOI:** 10.7759/cureus.46098

**Published:** 2023-09-27

**Authors:** Omobolanle A Obajeun, Abdelrahman Abaza, Arturo P Jaramillo, Faten Sid Idris, Humna Anis Shaikh, Ilma Vahora, Kiran Prasad Moparthi, Majdah T Al Rushaidi, MeghanaReddy Muddam, Pousette Hamid

**Affiliations:** 1 Pediatrics, California Institute of Behavioral Neurosciences and Psychology, Fairfield, USA; 2 Pathology, California Institute of Behavioral Neurosciences and Psychology, Fairfield, USA; 3 General Practice, California Institute of Behavioral Neurosciences and Psychology, Fairfield, USA; 4 General Surgery, St. George's University School of Medicine, Chicago, USA; 5 General Practice, Sri Venkata Sai (SVS) Medical College, Hyderabad, IND; 6 Psychology, California Institute of Behavioral Neurosciences and Psychology, Fairfield, USA; 7 Neurology, California Institute of Behavioral Neurosciences and Psychology, Fairfield, USA

**Keywords:** congenital torticollis, neck tilt, facial asymmetry, sternocleidomastoid muscle (scm), cervical spine anomalies, spinal deformiites, torticollis in children

## Abstract

Congenital torticollis is an abnormal tilt of the neck in a newborn especially on the side of the pathology with the chin pointing toward the contralateral side. The most frequent cause is termed congenital muscular torticollis (CMT) which is a structural abnormality in the muscle of the neck called sternocleidomastoid muscle. There are also other causes of congenital torticollis that may arise such as anomalies of the cervical vertebrae, syndromic causes, and ocular defects. Diagnosing these other causes of congenital torticollis requires careful examination, cervical X-ray, CT scan, and MRI. The objective of this review is to create an awareness of the different types and causes of cervical spinal deformity. It also confirms that it is easy to misdiagnose these rarer causes of congenital torticollis as seen in a clinical vignette of a newborn who was managed for CMT for about one year with physical therapy and later turned out to have an associated hemivertebrae and fusion of the second and third cervical vertebrae. It is rare but it has the burden of huge financial and psychosocial impact.

## Introduction and background

“Torticollis might have a lot of twists, tilts, turns, and troubles, but there is always a ray of hope.”

Globally, the prevalence of congenital torticollis averages between 0.3% and 1.9% [1]. According to some other research, out of every 250 newborns, there would be at least one incidence of congenital torticollis [1]. Hip dysplasia and the calcaneovalgus feet are the leading congenital orthopedic anomalies. Congenital torticollis follows them closely in terms of prevalence [1]. Congenital muscular torticollis (CMT) is, within the possible differential diagnoses, the most common type of congenital torticollis, with an incidence of 0.3-3.92% and a male-to-female ratio of 3:2.4.4 [2].

It is possible that a congenital torticollis patient also suffers from clubfoot and developmental dysplasia of the hip (DDH) as associated issues. However, in a set of DDH patients, only 1.9% of patients were reported to have clubfoot which means that the association between DDH, clubfoot, and CMT is not significant [3].

Torticollis is easily noticeable from outside the body which is why it is different from other many health conditions that develop silently from inside the body. It is associated with neck twisting and facial disorientation [4]. CMT is caused by the shortening of the sternocleidomastoid muscle, which may lead to neck movement limitation and craniofacial deformity [4,5]. The cosmetic problems on the face and neck can have a lifelong impact on a patient’s self-esteem [5]. Torticollis can come from birth and it can be acquired [4], and while cranio-facial irregularities are associated with congenital torticollis, they are not common with acquired torticollis [4]. Skeletal dysplasia and cervical spine anomalies are often found together in young patients, including connective tissue diseases [6].

However, more misleading is the silent torticollis that is due to cervical deformity and misleads physicians and caregivers in its early stage of presentation. Our patient’s case was initially managed as a case of CMT, and after 12 months of nil improvement, further radiological investigations were requested, and this turned out to be cervical spinal deformity. Most anomalies of the cervical spine are often detected until late childhood or adolescence [7]. The spine's unique anatomical structure makes it stand out among all the different vertebrae. The distinctiveness of the cervical spine is made more important by how it is formed from compounded processes [8]. Vertebrae fusion, segmentation anomaly, and cranio-cervical instability are some examples of cervical spine disorders [8]. This means that some can be simple without any clinical significance, while some can be complex and associated with neurologic and structural implications [7].

Before definitive treatment, parents and the affected child in this review admitted to having some level of social stigma. Our patient also had some isolation and social restrictions from some childhood activities that may cause strain on the neck. Isolation from peers in school, the exorbitant cost of treatment for surgical intervention options, the availability of few facilities that have multidisciplinary specialists to carry out surgical intervention, and the possibility of permanent neurologic injury and disability, if definitive treatment options cannot be accessed, are some of the concerns of the parents.

Case presentation

**Figure 1:**
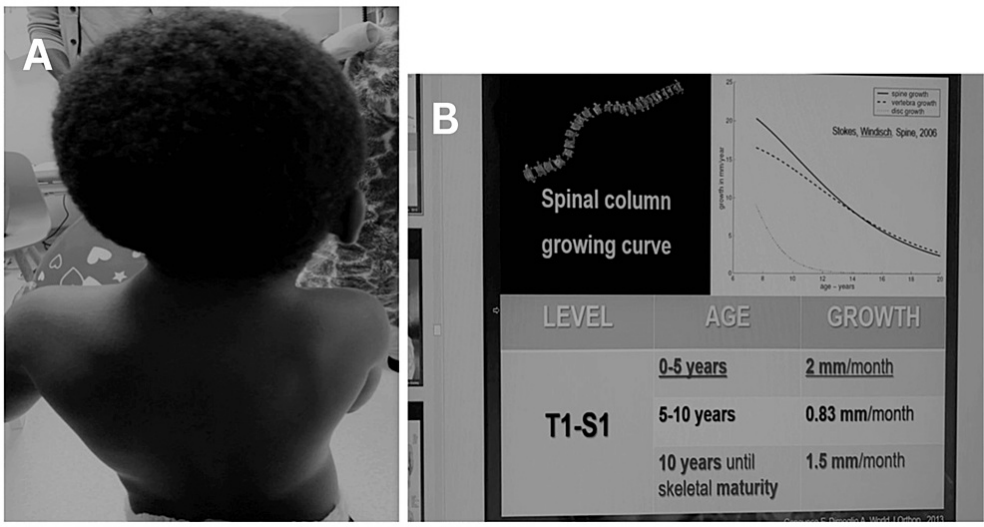
(A) The posterior view of the child after three years of physiotherapy. (B) The calibration reference for the curvature

A one-month-old male infant presented with a left-sided neck tilt in the pediatric outpatient department of the General Hospital, Ifako-Ijaiye, Nigeria. The parents had just returned to Nigeria following the delivery of their baby in the United States of America. History revealed that parents noticed the tilt from birth but assumed it was due to poor neck control of the newborn which they assumed to be a normal finding. At birth, the parents claimed that the pediatrician who reviewed the newborn did not observe any deformity, but they became worried due to the persistent one-sided neck tilt that was not resolving (Figure 1).

**Figure 2:**
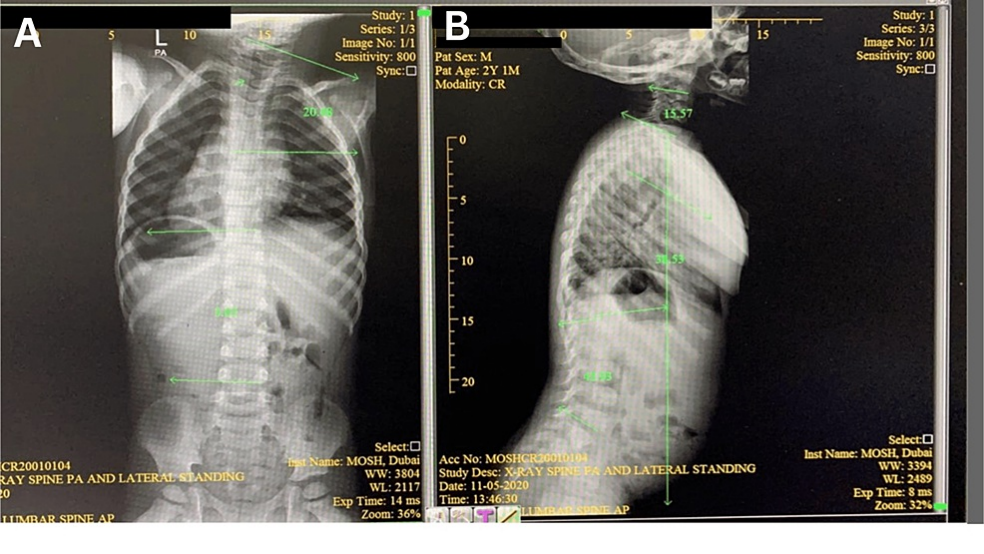
(A) Anterior and (B) lateral view of the X-ray images at three years old

Pregnancy was term and there was a history of prolonged labor at birth. Delivery was via emergency cesarean section. On physical examination, plagiocephaly facial asymmetry with fullness was observed more on the right than the left. The head tilted to the left, and the chin turned toward the right. A lump of mass was palpated on the left sternocleidomastoid muscle with associated muscle shortening. There was a limited rotation of the neck toward the left side. No other congenital abnormalities were found. A referral was given to commence physiotherapy sessions (Figure 2).

**Figure 3:**
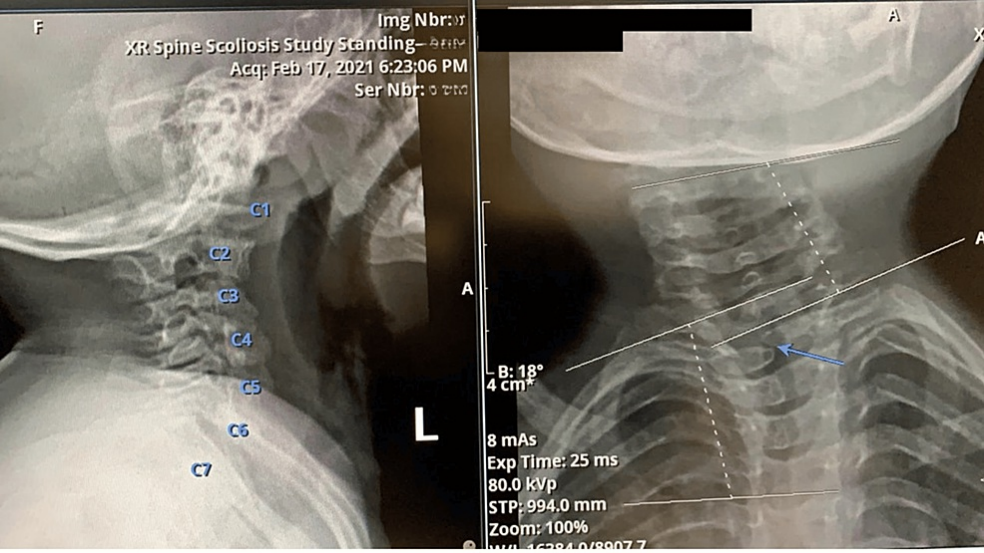
A closer view of the X-ray images, showing the C2 and C3 butterfly-shaped fusion

On review of the child after one year, neck tilt was still present despite consistent physiotherapy sessions and has developed more pronounced facial asymmetry. The child was referred for correction of plagiocephaly, but there was no facility available for such correction in Nigeria; hence, they traveled back to the United States of America. Unfortunately, correction with a head helmet could not be done due to the fusion of all the cranial fontanelles as a result of late presentation. Further investigations were ordered which included neck ultrasound, neck X-ray, cervical CT scan, and MRI. The review of the result showed butterfly-shaped cervical spine deformity affecting C2 and C3, and a referral to a neuro-spinal surgeon was advised (Figure 3). The final plan is to have surgical intervention for the correction of the cervical deformity at the age of five.

## Review

From the embryological point of view, the sub-axial cervical region and the upper segment of the spine are not the same, especially with respect to the configuration of the region between the occiput and C2-C3 disk space [8]. The radiographs from the investigation of the case in this report also validate this assertion. What this means is that in some instances, an anomaly of the upper cervical spine may lead to early symptoms in life because of the tendency to develop segmental instability and consequent spinal canal encroachment that may result in neurological deficits [8]. However, degenerative arthritis close to the region of synostosis and present early in adulthood can make any anomaly at the lower cervical region that extends from C4 downward to become symptomatic [8]. It is also noted that some cervical anomalies are discovered incidentally [9].

There are seven cervical vertebrae, C1 to C7, which are the key components of the spine, and the spine itself is segmented into the cranio-cervical junction (CCJ) as well as the sub-axial region [10]. The atlas C1 and the axis C2 are the two most cephalad vertebrae, and together with the occiput form the CCJ [10]. C3-C5, which are the most caudal vertebrae, form the sub-axial spine [10]. The rotation of the head and neck is hinged on the cervical spine, specifically in C1-C2, which also carries the weight of the cranium [10,11]. For the patient in this case report, the rotation of his neck is limited due to the irregular segmentation of C2 and C3.

The etiology of congenital torticollis remains unknown, but different theories have been proposed. The common types that are mentioned are ischemia, trauma during childbirth, and intrauterine malposition due to pelvic shape [1,12]. Intrauterine deformation has been proposed to be the commonest, and this occurs commonly in scenarios linked to uterine compression as well as insufficient intrauterine space and shortage of amniotic fluid, which is prevalent among first pregnancies [1,13]. For example, the mother of the patient in this case clarified that for a period of four to five months, there was no change in the position of her baby in the uterus, which suggests an intrauterine malposition. More so, it was her first pregnancy. Hence, the above theories are largely consistent with the findings in this case report.

A one-sided atlanto-occipital fusion and the lack of sternocleidomastoid, positional defect, Klippel-Feil syndrome (KFS), and pterygium colli, as well as vertebral disorders [1,14], hemivertebrae, Goldenhar syndrome, neurofibromatosis type 1, and iatrogenic and non-iatrogenic cervical deformity, are other causes of congenital torticollis [14].

Syndromes associated with cervical spine deformity include Down syndrome, Morquio syndrome [15,16], chromosomal number 5 syndrome [17], Kabuki syndrome [17-19], Noonan syndrome (Turner-like syndrome) [17,20,21], Aarskog syndrome [17,22], cervico-oculo-acoustic syndrome (Wildervanck syndrome), Müllerian duct and renal and cervical vertebral defects association, Jarcho-Levin syndrome (spondylothoracic dysplasia), proteus syndrome, arteriohepatic dysplasia, Gorlin syndrome, fibrodysplasia ossificans progressiva syndrome, spondylo-carpo-tarsal synostosis syndrome, multiple synostosis syndrome, Coffin-Lowry syndrome [17], rickets, achondroplasia, and diastrophic dysplasia [7].

The other acquired causes of torticollis are traumatic conditions, infections, inflammation of adjacent structures such as juvenile rheumatoid arthritis, tumoral conditions, and ocular and neurological dystonia [6].

In pediatric cases, CMT is the leading type of torticollis [1], and it is closely linked to the deformities of the sternocleidomastoid muscle [1,13]. However, this is not the case for the patient in this report, though it was wrongly diagnosed as CMT in the early stage as a lump of mass and was palpated on the left sternocleidomastoid muscle with associated muscle shortening. This anomaly is associated with endomysial fibrosis and collagen deposition which leads to the accumulation of fibroblasts around the muscle fibers and muscle atrophy [23,24]. Often, ipsilateral inclination and heterolateral rotation are seen in patients [1,25-27]. This may manifest early in the neonatal period or after birth [28].

Torticollis due to cervical spine anomaly is diagnosed when there is cervical malalignment in the coronal plane as a result of irregular formation or segmentation of the vertebra as it was seen in the radiographs of the child in this report. The lateral curvature of the spine is due to irregular vertebral formation [14,29,30]. It is tough to examine congenital osseous torticollis physically or via X-rays especially when patients do not have associated congenital anomalies [8,14,31]. Participation in sports or manipulation during anesthesia can result in fatal paralysis if such disorders are not diagnosed on time [17]. For these reasons, it is quite easy to overlook osseous torticollis in cases with undetectable congenital defects accompanying vertebral deformities, and examples of such include radial and renal dysplasia syndrome, imperforate anus, and tracheoesophageal fistula [31].

When the upper cervical region is invaginated upward into the foramen magnum, then Basilar impression is created which leads to the contraction of the components of the posterior cranial fossa because the components are housed by the tentorium over it [6]. Neurological damage from accidents or constriction of CSF flow are some of the risks associated with this [17]. This can be either primary or secondary. In the primary basilar impression, there are other associated anomalies such as atlanto-occipital fusion, hypoplasia of the atlas, bifid posterior arch of the atlas, odontoid abnormalities, KFS, and Goldenhar syndrome [17]. In the secondary basilar impression, there is softening of the bone, which leads to deformity developing later in life [7]. KFS is one of the syndromic causes of cervical deformity in which there is a vertebral fusion [31]. It can involve two segments: congenital block vertebrae or the entire cervical spine. It occurs between weeks 3 and 8 of life due to irregular segmentation of the cervical somites [7]. The etiology is still unknown except in some patients with inherited conditions [17]. Patients have features such as heart abnormalities, spina bifida, low hairline, facial plagiocephaly, scoliosis, and genitourinary problems [31]. According to some studies, while adult patients might present degenerative signs as a result of progressive cervical fusion, pediatric KFS patients did not present any signs [29].

Hemivertebrae is a congenital deformity that occurs as a result of failure of segmentation in one of the two sides of the vertebral body [29,32]. This condition presents just a useful disc in a well-formed cervical segment that is carrying hemivertebrae [29], leading to severe limitation of the activity of the affected segment. There is an annual increasing rate of tilt above the hemivertebrae of 2.5°, and compensatory curves below the hemivertebrae are often observed [29,33]. This is consistent with the curvature rate of the child in this report. The deviation of the child's neck started at 18o at the age of one and has progressed at the rate of 3o every year.

It is a usual occurrence to find deformities in the occipito-cervical region due to its complex formation mechanism [8]. Either a partial or complete fusion of the atlas at the rear end of the occiput can lead to occipito-cervical synostosis [17]. This is a disorder that is also called atlas occipitalization or the absorption of the atlas into the occipital bone [17]. Developmental irregularities of the occipito-cervical junction are often seen with Basilar impressions as well as odontoid deformities and are found equally in both male and female pediatric patients [17].

Atlantoaxial instability can occur, and this is common in the pediatric population due to the ligamentous laxity in this axis such as seen in Down syndrome. Furthermore, hypoplasia of the odontoid can occur such as in Morquio syndrome and spondyloepiphyseal dysplasia. There can be infection affecting the C1-C2 articulation at the odontoid and anterior side of C1 as seen in juvenile rheumatoid arthritis [6,15,16]. The hypoplastic posterior arch of C1 is usually seen with instability of C1-C2 [6]. This typically leads to a shortened sub-axial cervical spine, and, hence, patients are vulnerable to neurologic anomalies [6]. Anomalies of the odontoid process can also lead to atlantoaxial instability. This spans from partial hypoplasia to complete aplasia (absence) to disengagement of the dens from the axis [17]. As a result, there might be a neurological deficit and even death [4,7].

Abnormal facet complex, cervical rib, unfused spinous process, and vertebral fusion are other various anomalies that can be picked up on the subaxial view of CT scan sections [8]. On the coronal view, you can pick ossiculum terminale, paracondylar process/accessory occipito-cervical articulation, and assimilation. While on the sagittal view, you can see the prebasioccipital arch, C2 isthmus and internal height for high riding vertebral artery, ponticulus posticus, accessory ossicle of the atlas, abnormal ossicle of the atlas, and the abnormal facet complex [8].

Management of any form of cervical spine deformity presenting with torticollis starts from detailed history taking, thorough examination including ophthalmologic evaluation, and investigation using ultrasonography and MRI [4]. This is to enable the development of the right treatment plan as oftentimes, most interventions are age-limited. For instance, the child in this report missed the possibility of having his facial plagiocephaly corrected because the right diagnoses were not done on time.

Positioning, for example, rotation of the chin in the direction of the tilt, flexion, and stretches are initial necessary treatments in the absence of congenital anomalies of the cervical spine [1]. These initial interventions should be done regularly during the week [1]. Clearly, the most essential initial treatment is stretching methods, especially in CMT cases [1,26,34].

For pediatric patients, the radiographic appearance of the cervical spine can give an accurate interpretation of images and examination of the patients and most importantly because the ossification centers are the main concern areas for the treatment of non-muscular causes of torticollis [6]. However, it can be difficult to interpret radiographs when there is incomplete ossification of the cervical spine, especially in pediatric patients.

For progressive cervical deformity, especially when the patient is in a constant painful condition and nerve roots are being compacted, surgical intervention should be considered [29]. The case of the child in this report presents constant complaints of pain in the neck region, though with no reported nerve compaction, hence, the need for surgical intervention. Importantly, osteotomy and level of flexion are closely related to the effect of any surgical treatment [29,35,36].

## Conclusions

Parents and caregivers may not fully understand or expect the gravity of the outcome of their child’s neck tilt once CMT has been ruled out. Hence, it is good to carry them along from the onset of their first contact with healthcare and let them know of the different causes of congenital torticollis. Once a correct diagnosis is made at the earliest possible period, it can help to initiate proper treatment modality and, hence, avert and limit the complications that may likely occur, such as facial asymmetry and disfigurement, scoliosis, neck pain and limb pain, and functional impairment such as instability risk and spinal cord encroachment. The cost of pursuing unnecessary physical therapy will be avoided. Parents may struggle to come to terms with this new diagnosis when it is diagnosed late especially if they are not properly educated, which may cause delay in seeking definitive treatment.
